# A rare morphological gem in fulminant meningococcal sepsis

**DOI:** 10.1016/j.bjid.2026.105822

**Published:** 2026-05-02

**Authors:** Martin P. Dieterle, Stefano Malvestiti, Barbara Zieger, Hans Fuchs, Daniel Matheisl

**Affiliations:** a University of Freiburg, Faculty of Medicine, Medical Center, Division of Neonatology and Paediatric Intensive Care Medicine, Department of Pediatrics and Adolescent Medicine, Freiburg, Germany; b University of Freiburg, Faculty of Medicine, Medical Center, Division of Pediatric Hematology and Oncology, Department of Pediatrics and Adolescent Medicine, Freiburg, Germany

A 4-month-old girl presented to our pediatric emergency department with septic shock requiring fluid resuscitation and vasoactive support. Laboratory findings suggested severe bacterial infection with a procalcitonin level of 249.5 ng/mL (reference < 0.05 ng/mL). Empirical antimicrobial treatment with piperacillin/tazobactam was instantly initiated.

The patient exhibited progressively enlarging ecchymoses consistent with purpura fulminans. Cefotaxime and vancomycin were therefore immediately added, given the suspicion of meningococcal sepsis. Markedly elevated D-dimers (>35 mg/L), thrombocytopenia (nadir 37 G/L) and severely reduced protein-C activity (14%) confirmed the presence of Disseminated Intravascular Coagulation (DIC), further supporting the suspected diagnosis. Following transfusions of platelets, fresh frozen plasma and protein-C concentrate, coagulation parameters eventually normalized.

At presentation, a peripheral blood smear was prepared for morphological evaluation of leukocytes and revealed the findings shown in Fig. 1. Intraleukocytic and peri-/intraerythrocytic cocci and diplococci were identified. Further microbiological PCR-diagnostics confirmed invasive *Neisseria meningitidis* (serogroup W). The patient recovered completely.

**Fig. 1:**
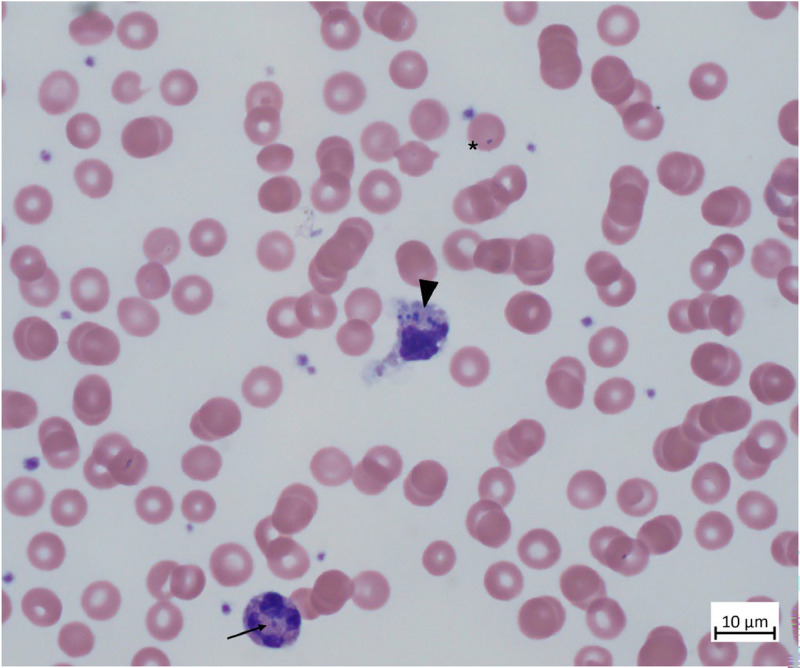
Peripheral blood smear of a 4-month-old girl (black arrow probably indicating the inactivated X-chromosome [“Barr body”]) with meningococcal sepsis. The exceedingly rare findings of intraleukocytic cocci and diplococci (black arrowhead) and peri-/intraerythrocytic diplococci (black asterisk) are depicted. Microbiological PCR-diagnostics confirmed invasive infection with Neisseria meningitidis serogroup W.

Intraleukocytic detection of meningococci in peripheral blood smears remains a rare finding.^1^ A direct correlation between clinical severity/bacterial load and the detectability of meningococci in peripheral blood smears has not been established yet.^2^ However, if present, this finding may precede definitive microbiological results by days.

*Neisseria meningitis* can bind to erythrocyte Complement Receptor 1 (CR1) on the surface of red blood cells, facilitating evasion of phagocytosis by leukocytes and favoring meningococcal survival within the bloodstream. Visualization of meningococci adjacent to or within erythrocytes is exceedingly rare.^3^ Mechanistically, CR1 acts as an inhibitor of the classical complement cascade by serving as a co-factor for factor I, which cleaves C3b and C4b into inactive forms, thus preventing formation of the membrane attack complex and subsequent bacterial lysis.^4^

In ambiguous cases, a cost-effective peripheral blood smear can complement clinical findings in septic shock and guide therapeutic decisions.

## Data availability

The data that support the findings of this study are available from the corresponding author upon reasonable request.

## Conflicts of interest

The authors declare no conflicts of interest.
